# Cardiovascular adaptations in judo: a narrative review

**DOI:** 10.3389/fspor.2025.1607549

**Published:** 2025-06-18

**Authors:** Carlo Rossi, Valerio Giustino, Antonino Patti, Roberto Roklicer, Marko Manojlovic, Tatjana Trivic, David Fukuda, Antonino Bianco, Patrik Drid

**Affiliations:** ^1^ Sport and Exercise Sciences Research Unit, Department of Psychology, Educational Science and Human Movement, University of Palermo, Palermo, Italy; ^2^ Faculty of Sport and Physical Education, University of Novi Sad, Novi Sad, Serbia; ^3^ Faculty of Education, Free University of Bozen-Bolzano, Brixen-Bressanone, Italy; ^4^ Institute of Exercise Physiology and Rehabilitation Science, University of Central Florida, Orlando, FL, United States

**Keywords:** aerobic capacity, cardiac function, cardiac hypertrophy, heart rate variability, hemodynamic responses, judo, martial arts, combat sports

## Abstract

Judo is a high-intensity combat sport requiring substantial aerobic and anaerobic capacity. Although research has explored the physiological demands of different sports over the years, few studies have investigated the specific cardiovascular adaptations that occur in judoka. This narrative review examines these adaptations by focusing on cardiac function, heart rate variability (HRV), and hemodynamic responses, with the aim of summarizing the effects of judoka training on cardiovascular health and the relationship with athletic performance. Judo training improves aerobic capacity, with VO_2_max values similar to those of team sports athletes. It stimulates physiological hypertrophy of the left ventricle, improving cardiac function. Autonomic regulation shows a parasympathetic predominance, indicating better stress adaptation. Vascular adaptations include increased arterial elasticity and optimal blood pressure management, with judoka exhibiting lower blood pressure values than the general population. In summary, these adaptations promote cardiovascular health and improve athletic performance, although monitoring is essential to prevent overtraining and long-term issues.

## 1 Introduction

Judo is a combat sport that combines technical skills, strength, endurance, and tactics, characterized by intermittent high-intensity efforts alternating with active or passive recovery phases (1). During a match, athletes must perform explosive movements, such as projections or throws, takedowns, and ground fighting phases (ne-waza), followed by recovery periods that vary according to the rhythm of the fight (2). This physiological model imposes a high stress on the cardiovascular system, determining specific adaptations that influence both athletic performance and the athlete's long term health (3).

Indeed, judo matches last on average about 5 min for men and 4 min for women, with possible extensions in the event of a draw. During a fight, athletes alternate short periods of explosive efforts, characterized by attacks and defenses, with periods of active or passive recovery. Studies have shown that the efforts-recovery ratio in judo is about 2:1, with high intensity phases lasting 20–30 s, followed by short pauses (4). Furthermore, the heart rate of judokas during a fight can reach values above of 180 beats per minute (bpm), demonstrating the high cardiovascular effort required (5).

The presence of repeated explosive actions with short recoveries in judo determine a significant oxygen consumption and a frequent activation of anaerobic lactic metabolism (6). During a match, the heart rate can reach and maintain between 80% and 95% of the maximum heart rate, with peaks close to 100% in moments of maximum effort (7). This implies a high cardiac and metabolic demand, with adaptations that include increased cardiac output, greater oxygen extraction capacity by the muscles and improvements in tolerance to metabolic stress (8).

Hence, cardiovascular adaptations in combat sports such as judo are of particular interest to the scientific community, as these sports require a unique combination of aerobic and anaerobic capacity (9, 10).

Furthermore, recovery management is crucial to optimize performance and prevent fatigue, with strategies such as cold water immersion having been shown to be effective in improving post-match recovery (11). These data support the need for targeted training to improve athletes' recovery capacity and endurance, ensuring optimal performance during competition.

### 1.1 Cardiovascular adaptations in judo training

Judo training induces numerous cardiovascular changes, with some peculiarities due to the intermittent nature of the sport (12). For example, studies conducted on elite judoka have shown significant increases in maximal oxygen consumption (VO_2_max), indicating a measurable improvement in aerobic capacity (13). A high VO_2_max is associated with a better ability to maintain high work intensities and a faster recovery between explosive actions (14). However, judo-specific training enhances the ability to tolerate and effectively use lactate as an energy source, delaying fatigue and improving performance during high-intensity efforts (7).

Another key aspect is the improvement of heart rate recovery (HRR), an indicator of the efficiency of the autonomic nervous system in restoring the physiological balance post-exertion. Well-trained judo athletes show a more rapid reduction in heart rate after intense exercise, suggesting enhanced vagal control and more efficient recovery compared to less trained judo athletes or practitioners of lower-intensity intermittent sports (15).

Heart rate variability (HRV), another key parameter for assessing cardiovascular health and recovery capacity, is improved in elite judoka compared to amateur practitioners (12). This implies a greater adaptability of the autonomic nervous system to physical efforts, reducing the risk of overtraining and improving blood pressure regulation (16).

### 1.2 Health and performance implications

Cardiovascular adaptations induced by judo not only improve athletic performance but also have positive effects on general health. Regular practice of high-intensity interval sports has been associated with a reduction in the risk of cardiovascular disease, thanks to improvements in endothelial function, blood pressure regulation, and insulin sensitivity (15). Furthermore, high-intensity training such as that of judo stimulates the production of vascular growth factors, improving vasodilation and the ability to transport oxygen to tissues (17). Physiological adaptations resulting from judo training contribute to athletes' overall health (18).

However, in addition to the cardiovascular benefits, it is essential to consider the weight management strategies adopted by judokas to fit into the established weight categories for competition (19). Weight management is a crucial aspect of judo training, but it can carry to significant risks if not performed in a controlled manner. Athletes often resort to rapid weight loss (RWL) methods, which include caloric restriction and dehydration (20). Athletes who undergo to RWL can lead to stress to the cardiovascular system (19, 21). In fact, RWL can temporarily reduce plasma volume and increase resting heart rate, which may have negative consequences on performance, long-term cardiovascular function (22), and metabolic health (23).

A previous study have shown that safer weight management strategies, such as evidence-based weight management programs, can reduce the risks associated with extreme weight loss (19). For this reasons, coaches and trainers should adopt safe and effective weight management strategies, minimizing negative effects on cardiovascular function and optimizing post-competition recovery (19).

Based on these knowledges, the aim of this narrative review is to examine the physiological adaptations induced by judo practice, with a focus on cardiac function, HRV, and hemodynamic responses, to understand how training and competition can influence the cardiovascular system of judo athletes, and to examine the relationship with athletic performance.

This narrative review is divided into subsections. The first section examines the cardiovascular demands and physiological responses to judo training, detailing its impact on aerobic and anaerobic capacity, cardiac function, and vascular health. The second section synthesizes existing studies that evaluate cardiovascular training strategies, assessing their effectiveness in enhancing performance and reducing fatigue in judo athletes.

## 2 Materials and methods

To retrieve eligible articles, a manual search was conducted on the following databases: PubMed, Google Scholar, and Web of Science. To identify suitable articles, the following search strategy was adopted: (Cardiovascular effects) AND (judo) OR (combat sports); (Cardiovascular health) AND (judo athletes) OR (judo training). This search was extended using the bibliography within the recruited texts. Articles published in English were considered.

A narrative review approach was chosen to present and explain the current understanding of cardiovascular aspects in judo. Unlike systematic reviews or meta-analyses—planned as future steps—this format allows for an exploratory discussion, highlighting emerging trends, key findings, and gaps in the literature. By mapping these physiological adaptations, this review provides valuable insights for coaches, sports scientists, and athletes, guiding evidence-based training strategies to optimize cardiovascular health and performance in judo.

## 3 Results

The literature review highlighted several significant cardiovascular adaptations in judokas, with positive effects on both performance and health. The studies included indicated improvements in aerobic and anaerobic capacity, heart morphological changes, enhanced autonomic regulation, and improved vascular function and blood pressure.

**Figure 1:**
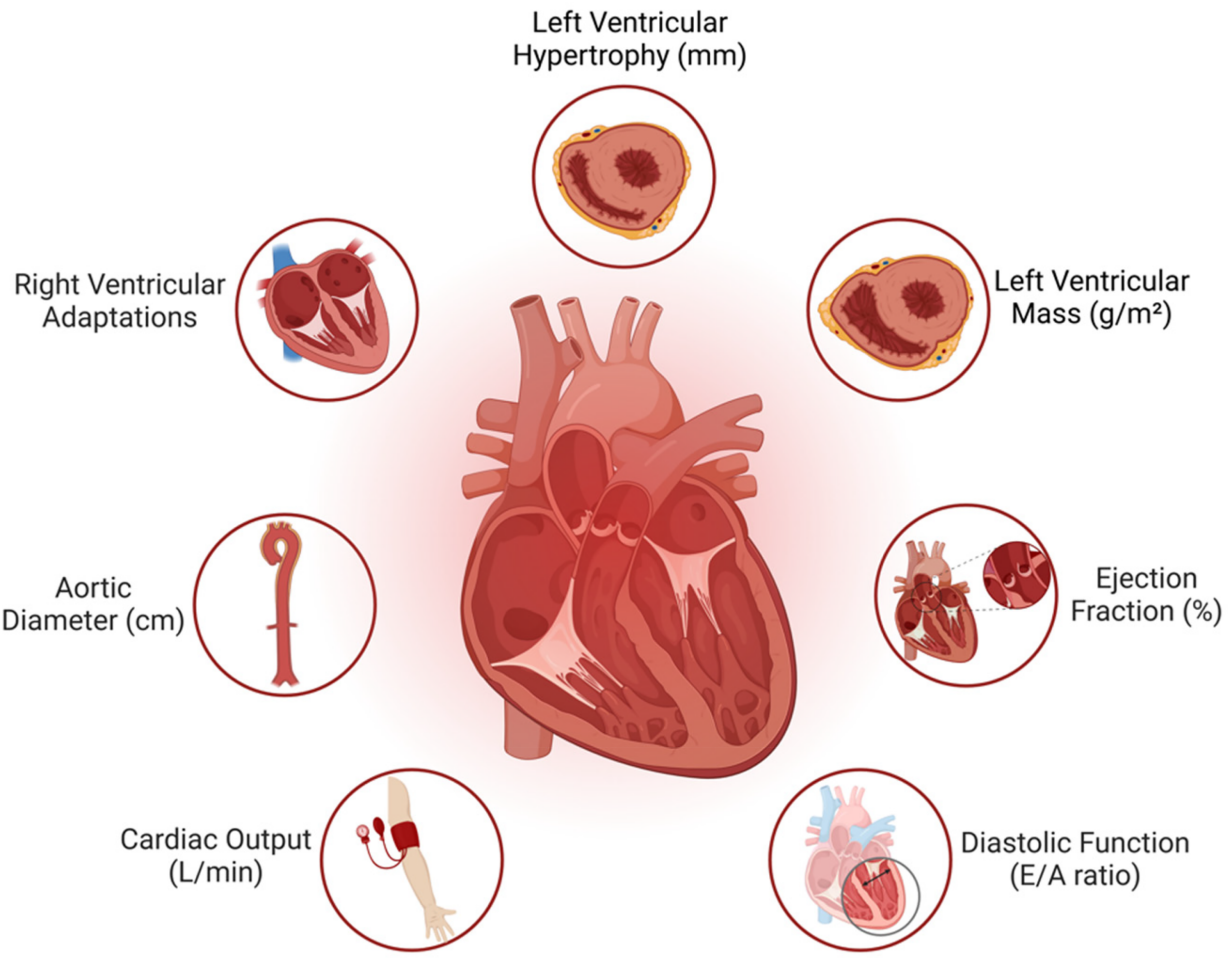
Morphological and physiological heart changes in judokas.

The main indicators of cardiovascular response in judokas are reported in Table 1, and the heart morphological and physiological adaptations in judokas are reported in Table 2 and Figure 1.

**Table 1:** Indicators of cardiovascular response in judokas.

Parameter	Value in judokas	Comparison with other disciplines	Study
VO_2_max (ml/kg/min)	50–60	Similar to soccer and basketball players	Franchini et al. (49)
Heart Rate Recovery (HRR)	Faster	Better than less intense intermittent sports	Buchheit et al. (41)
Post-exercise Lactate (mmol/L)	Higher, but cleared faster	Better metabolic acidosis regulation	Bonato et al. (50)
HRV (RMSSD, HF)	Increased	Higher parasympathetic activity vs. sedentary	Morales et al. (51)
Maximum Heart Rate (% HRmax)	80–95%, with peaks at 100%	High cardiovascular response to effort	Franchini et al. (38)
Resting Blood Pressure	Lower than the general population	Similar to endurance sports	Kumagai et al. (52)
Endothelial Function	Improved	Greater nitric oxide production	Bescos Garcia. (53)

VO_2_max, maximal oxygen consumption; HRR, heart rate recovery; HRV, heart rate bariability; RMSSD, root mean square of successive differences; HF, high frequency power.

**Table 2:** Morphological and physiological adaptations of the heart in judokas.

Parameter	Value in judokas	Comparison with other disciplines	Study
Left Ventricular Hypertrophy (mm)	10–14	Similar to endurance athletes	Pelliccia et al. (54)
Left Ventricular Mass (g/m^2^)	120–140	Comparable to rowing and cycling	Fagard. (55)
Ejection Fraction (%)	55–70	Within normal range for athletes	Pluim et al. (56)
Diastolic Function (E/A ratio)	1.2–1.8	Better filling capacity than sedentary individuals	D'Andrea et al. (57)
Right Ventricular Adaptations	Enhanced	Larger dimensions compared to non-athletes	La Gerche et al. (58)
Aortic Diameter (cm)	3.2–3.8	Adaptive response similar to endurance training	Sharma et al. (59)
Cardiac Output (L/min)	12–20	Greater stroke volume and efficiency to sedentary	Dittrich et al. (60)

### 3.1 Aerobic and anaerobic capacity

Judo training leads to an increase in both aerobic and anaerobic capacity, enabling athletes to sustain intense and repeated efforts without rapid fatigue.

• **Maximum oxygen consumption (VO_2_max):** According to Franchini et al., elite judokas have mean VO_2_max values between 50 and 60 ml/kg/min, values higher than the sedentary population and comparable to those of athletes of team sports such as football or basketball. A higher VO_2_max is associated with better oxygen transport and greater resistance to prolonged effort (7).
• **Post-exercise recovery efficiency:** The time required for HRR after exercise is considered an indicator of cardiovascular health. A previous study has shown that judokas have a faster reduction after intense exercise compared to non-athletes, suggesting greater cardiovascular efficiency. This improvement is attributed to increased vagal tone and optimized aerobic recovery capacity (24).
• **Lactate production and disposal:** Well-trained judokas show a greater tolerance to metabolic acidosis, with higher lactate levels but faster clearance times than other athletes (25). This suggests a greater efficiency in the regulation of anaerobic metabolism, essential to sustain repeated explosive efforts (26).

### 3.2 Heart morphological changes

Judo training induces to morphological and physiological adaptations similar to those found in endurance and strength sports.

• **Left ventricular hypertrophy:** Several studies have reported that judokas exhibit an increase in left ventricular mass, a common physiological adaptation in athletes in sports with high cardiovascular demand (27, 28). This increase in ventricular mass allows for a greater stroke volume, improving the efficiency of oxygen transport during intense physical activity (29).
• **Increased thickness of the interventricular septum:** Although the morphological changes of the heart in judokas are similar to those found in endurance sports, such as cycling or swimming, judokas show an increased thickness of the interventricular septum, a typical characteristic of power sports (30). This reflects the combination of aerobic and anaerobic components typical of judo.

### 3.3 Autonomic regulation

The balance between the sympathetic and parasympathetic nervous systems is crucial for recovery and performance in high-intensity sports. Studies indicate that well-trained judokas show a predominance of vagal tone at rest, suggesting better autonomic control.

• **Heart Rate Variability (HRV):** It is known that judokas present significantly higher HRV values than sedentary subjects, with a predominance of parasympathetic activity at rest. This implies a better regulation of the cardiovascular system and a greater capacity to adapt to load variations (31).
• **Specific HRV indices:** A previous study reported that in judokas the Root Mean Square of the Successive Differences (RMSSD) and the High Frequency power (HF) indices, indicators of parasympathetic modulation, were higher than in control groups. This suggests a better post-exercise recovery capacity and a reduced predisposition to cardiovascular stress (32).
• **Effect of high-intensity training:** Previous studies have shown that high-intensity interval training (HIIT), often used in judo training programs, improves HRV and cardiovascular efficiency, reducing the risk of overtraining and improving the ability to handle repeated intense efforts (12).

### 3.4 Blood pressure and vascular function

Judo training also has positive effects on blood pressure and vascular function, contributing to the prevention of cardiovascular diseases.

• **Reduction of resting blood pressure:** It seems that judokas tend to present lower blood pressure values than the general population, thanks to increased arterial elasticity and reduced peripheral vascular resistance (33). This adaptation is similar to that observed in endurance athletes and represents a protective factor against hypertension.
• **Acute response to competition:** During a judo match, significant increases in blood pressure and heart rate are observed, with values that can reach 90%–100% of maximum heart rate (34). However, the return to basal levels occurs rapidly after the fight, suggesting an excellent cardiovascular recovery capacity in well-trained athletes.
• **Improvements in endothelial function:** High intensity training typical of judo stimulates the production of nitric oxide, improving vasodilation and endothelial function (35). This contributes to better regulation of blood pressure and an increase in the capacity to transport oxygen to the muscles during exercise.

## 4 Discussion

This narrative review aimed to examine the physiological adaptations induced by judo practice, with a focus on cardiac function, HRV, and hemodynamic responses, to understand how training and competition can influence the cardiovascular system of judo athletes, and to examine the relationship with athletic performance.

The analysis of cardiovascular adaptations in judo highlights how this sport, characterized by intermittent high-intensity efforts alternating with recovery phases, induces a series of specific physiological changes that optimize athletic performance and improve cardiovascular health. The results of this review confirm that well-trained judoka develop remarkable aerobic and anaerobic capacity, adapted cardiac morphology, enhanced autonomic regulation, improved vascular function, and blood pressure management. However, these changes are not without health implications, requiring a balance between performance and prevention of overtraining or long-term adverse effects.

### 4.1 Aerobic and anaerobic capacity

Improved aerobic and anaerobic capacity is one of the most important adaptations for judoka, who must sustain high-intensity efforts alternating with recovery periods. Research suggests that VO_2_max values between 50 and 60 ml/kg/min, such as those observed in judoka, are indicative of a high level of cardiovascular conditioning and endurance (36). Although these values are comparable to those observed in other team sports, it is important to emphasize that judo training does not focus only on endurance but also on explosive strategies, such as those needed to execute effective attacks and defenses.

The effects of HIIT on judoka, documented by a previous study, go beyond the simple improvement of aerobic capacity (37, 38). Such training also stimulates the ability to manage lactate accumulation, allowing athletes to maintain high performance despite increasing fatigue (39). The faster recovery of heart rate post-exertion suggests that judoka have a highly adapted cardiovascular response, which favors a rapid removal of metabolic waste products (36–38). However, the ability to manage metabolic acidosis and lactate management may be variable depending on the intensity and duration of training, as well as the experience of the athlete.

### 4.2 Heart morphological changes

Physiological hypertrophy of the left ventricle, observed in judoka, is a well-known adaptation in many high-intensity and endurance sports (28). The comparability of these changes with those found in endurance sports, such as cycling and running, suggests that judo stimulates a response of the heart that improves the efficiency of blood pumping (27). However, unlike purely aerobic sports, judo also requires significant anaerobic and strength work, which could explain the increased thickness of the ventricular walls (3). This could have direct benefits for the management of explosive efforts and for improving the ability to cope with high-intensity efforts during combat. This type of cardiac hypertrophy appears to be a beneficial adaptation for athletes, but attention must be paid to the risk of cardiovascular overload in the case of excessive training without adequate recovery periods (40). Regular monitoring of cardiovascular health may, therefore, be essential to prevent risks related to hypertension or heart failure in elite judoka.

### 4.3 Autonomic regulation

Adaptations in the autonomic regulation of the nervous system are one of the hallmarks of well-trained athletes. In the case of judoka, high parasympathetic activity at rest, indicated by HRV parameters such as RMSSD and HF, suggests a higher ability to handle stress and recover quickly from exertion. These autonomic adaptations are indicative of good cardiovascular health and a balanced response to load variations during training and competition (32, 41, 42).

The importance of autonomic regulation is particularly evident in judo, where phases of high intensity alternating with periods of active and passive recovery require a rapid transition between states of stress and recovery (41). The parasympathetic predominance observed in judoka could not only improve performance but also reduce the risk of cardiovascular disease, contributing to better stress management and the prevention of overtraining (43).

### 4.4 Blood pressure and vascular function

Another positive aspect of cardiovascular adaptations in judo is the improvement of vascular function, which translates into greater arterial elasticity and optimal management of blood pressure. Judokas tend to have lower blood pressure values than the general population, a protective factor that reduces the risk of hypertension and other cardiovascular diseases. However, transient variations in blood pressure and heart rate during combat are normal physiological phenomena, but it is important to monitor them to avoid long-term problems.

Various studies found that the ability to quickly return to baseline post-exercise is a sign of optimal cardiovascular health, but it is important that training is well-balanced to avoid periods of excessive stress on the cardiovascular system (44, 45).

## 5 Conclusions

Judo, as a high-intensity combat sport, involves a series of cardiovascular adaptations that improve athletes' performance and overall health. The adaptations observed in judoka, including improved aerobic and anaerobic capacity, physiological hypertrophy of the left ventricle, optimal autonomic regulation and improved vascular function, confirm the effectiveness of this sport in inducing positive changes in the cardiovascular system. In particular, the ability to recover quickly after intense stress and the resulting cardiovascular efficiency are indicators of well-balanced training. However, although cardiovascular adaptations are generally favorable, training sessions must be carefully planned to avoid long-term negative effects, such as overtraining or disorders related to excessive intensity. Continuous assessment of athletes’ cardiovascular health, combined with a periodized training program, may be the key to optimizing long-term benefits, ensuring high performance and preventing complications.

### 5.1 Practical implications and future research directions

The cardiovascular adaptations observed in judo not only improve the performance of athletes but also appear to have positive effects on overall cardiovascular health. However, it is essential to recognize that the intensity of judo training requires careful planning to ensure that the benefits are not offset by potential negative effects (46). To optimize cardiovascular health while maintaining peak performance, coaches should implement structured periodization, monitor HRV to assess autonomic balance, and ensure adequate recovery strategies. Additionally, safe weight management practices should replace RWL methods to prevent negative cardiovascular consequences (47, 48). By integrating these principles, judo training can provide long-term health benefits while reducing the risks associated with excessive training loads.

Future research could focus on evaluating the long-term effects of judo training, considering the relationship between intense sport practice and the prevention or development of cardiovascular diseases. Furthermore, it would be interesting to explore how personalized training strategies, which adequately balance aerobic and anaerobic efforts, can further optimize cardiovascular adaptations, minimizing the risks of overtraining or health problems. Furthermore, it would be optimal to try to perform post-career training to avoid adverse events once the athlete retires from competitive sports.
